# Identification of novel prognosis-related genes in the endometrial cancer immune microenvironment

**DOI:** 10.18632/aging.104083

**Published:** 2020-11-06

**Authors:** Jian Ma, Jing-Kai Zhang, Di Yang, Xiao-Xin Ma

**Affiliations:** 1 Department of Obstetrics and Gynecology, Shengjing Hospital of China Medical University, Shenyang 110004, China

**Keywords:** endometrial cancer, immune microenvironment, prognosis, CD74, CD52

## Abstract

The incidence of endometrial cancer is increasing each year, and treatment effects are poor for patients with advanced and specific subtypes. Exploring immune infiltration-related factors in endometrial cancer can aid in the prognosis of patients and provide new immunotherapy targets. We downloaded immune metagene and functional data of patients with different subtypes of endometrial cancer from The Cancer Genome Atlas database and selected the lymphocyte-specific kinase (LCK) metagene as a representative genetic marker of the immune microenvironment in endometrial cancer. The results showed that LCK metagene expression is related to the prognosis of patients with endometrioid endometrial adenocarcinoma subtypes and highly correlated with the *PTEN* and *PIK3CA* mutational status. A search for LCK-related modules returned seven independent genetic predictors of survival in patients with endometrial cancer. The TIMER algorithm showed that the expression of these seven genes was positively correlated with the infiltration levels of six types of immune cells. The diagnostic value of these markers was validated using real-time quantitative PCR and immunohistochemical methods. Our results identified CD74, HLA-DRB5, CD52, HLA-DPB1 and HLA-DRB1 as possible valuable genetic markers for the diagnosis and prognosis of endometrial cancer and provided a theoretical basis for immunotherapy targets for its clinical treatment.

## INTRODUCTION

Endometrial cancer (EC) is an epithelial malignant tumor that occurs in the endometrium. In 2019, an estimated 720,000 women living in the United States have been diagnosed with EC, and 54,000 cases bas be newly diagnosed [1]. According to recent statistics from the National Cancer Center of China, new cases of EC in 2015 ranked among the top 10 malignant tumors, accounting for 3.88% of all malignant tumors in women in China, increased from 3.79% in 2014 [2]. In the past ten years, because of the irregular use of hormones and changes in people’s living environment and lifestyle, the prevalence and mortality of EC have increased [3]. The treatment options for EC include surgery, radiotherapy and chemotherapy, hormone therapy, and targeted therapy [4]. For patients with advanced metastatic or recurrent EC, the rate of treatment failure remains high because of the lost opportunity for surgery [5]. Moreover, for specific EC subtypes, such as relapsed and endometrial serous carcinoma, the prognosis is especially poor [6]. Paclitaxel combined with carboplatin is the first-line treatment for advanced recurrent and metastatic EC. In addition, platinum drugs and megestrol acetate have been approved for the palliative treatment of advanced EC, but the therapeutic effect is very limited [7]. Studies have shown that 50% of Caucasian, 21.9% of Asian, and 12.5% of Pacific island populations show loss of expression of one or more mismatch repair genes [8]. Genetic polymorphisms in *TGFB1*, *TGFBR1*, *SNAI1* and *TWIST1* are associated with EC susceptibility in Chinese Han women [9].

From a pathological perspective, EC is a heterogeneous disease with widely variable clinical outcomes, both in terms of prognosis and treatment response. With the advent of the genetic era, EC has been divided into four molecular categories, namely POLE ultra-mutated, microsatellite instable (MSI), copy-number low/microsatellite stable (MSS), and copy number high/serous-like [10]. POLE-mutated and MSI EC have high mutation rates and stronger associations with immunogenic tumors. As such, immune checkpoint inhibitors such as PD1/PD-L1 antibody treatment can be used. In contrast, the copy-number low and copy number high types have lower mutation rates, are related to non-immunogenic tumors, and, in such cases, combined immunotherapy can be used to turn cold tumors into hot tumors [11–12]. Therefore, immunotherapy is a potentially useful treatment strategy for patients with advanced EC. Although some patients have achieved encouraging results with this intervention, some patients do not respond to immunotherapy [13]. PD-L1 antibody is widely approved for the treatment of MSI type EC, but the incidence of EC MSI is only approximately 20% and most patients have the MSS type. MSS EC is treated with PD-L1 antibody with a very low effective rate. These patients who have progressed after first-line treatment have very limited treatment options [14].

The tumor immune microenvironment is complex and diverse and may affect the growth of pre-cancerous cells, directly contrasting the immunotherapy of malignant tumors [15]. The immune microenvironment is an integral part of the tumor microenvironment (TME). It is mainly composed of tumor-infiltrating lymphocytes (TILs) and other immune cells that penetrate the tumor tissue. TILs mainly include T cells, macrophages, natural killer cells, and dendritic cells. As part of the cell-mediated immune response, TILs can lead to the clearing of tumor cells [16]. Stimulating the immune system and enhancing the anti-tumor function of the TME may be a novel approach for killing tumor cells and, to this end, researchers are investigating the combined use of various immunological checkpoint-based treatment strategies with targeted drugs, local area therapy, and other forms of immunotherapy [17]. EC cells can escape attack by the host immune system in various manners, such as self-modification and changes in the cell surface co-stimulation of molecular expression [18–19], which leads to changes in the composition and function of the immune microenvironment [20], ultimately leading to tumor immune escape. Reversing the immune escape of the tumor is an effective approach for inhibiting the progression of EC [21]. The immune escape mechanism in the TME of advanced EC is highly heterogeneous. Studies have shown that many immune cells often accumulate in and around EC tissues [22]. Furthermore, the presence of a large number of CD8^+^ T lymphocytes and CD45RO^+^ T lymphocytes is associated with an increase in the overall survival (OS) of patients with EC [23]. Therefore, exploring the factors associated with immune infiltration in EC may help evaluate the prognosis of these patients and provide new targets for immunotherapy.

In this study, we used a series of bioinformatics tools to determine the appropriate immune scoring method for different clinical subtypes of EC in The Cancer Genome Atlas (TCGA) database. We identified possible correlations between gene expression in the immune microenvironment of EC and prognosis. We verified this expression in EC and normal tissues and analyzed the relationship between expression and the disease-free survival rate. Finally, we identified several genes as possible immune microenvironment indicators of prognosis in EC, as well as possible targets for immunotherapy.

## RESULTS

### Selection of the lymphocyte-specific kinase (LCK) metagene as a representative genetic marker in the immune microenvironment of EC

**Figure 1:**
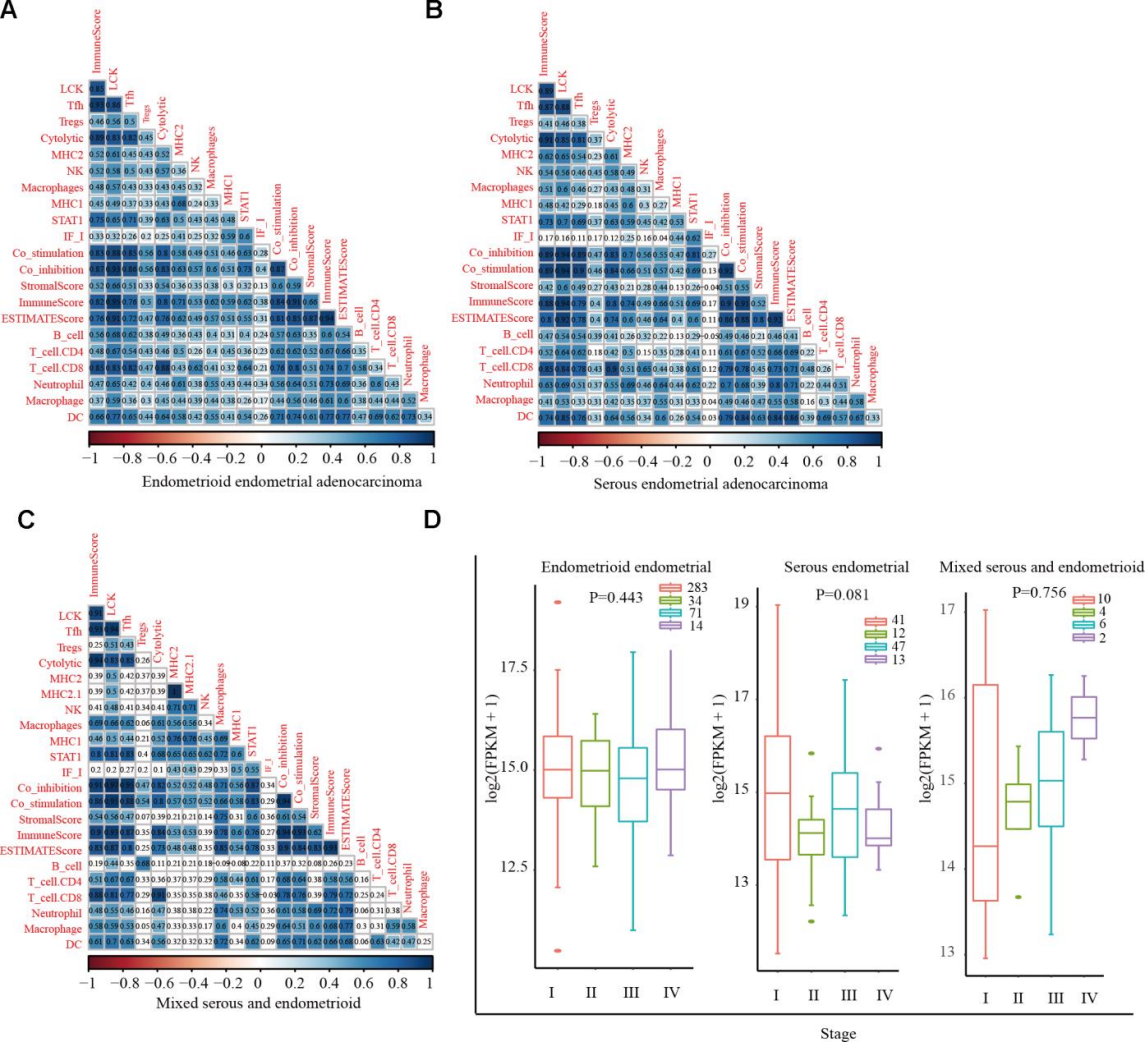
**Correlations between different immune scores in patients with different endometrial cancer subtypes.** (**A**) Positive correlation between LCK metagene score and other types of immune-related scores in endometrioid endometrial adenocarcinoma (cor = 0.84). (**B**) Serous endometrial adenocarcinoma (cor = 0.83). (**C**) Mixed serous and endometrioid (cor = 0.85). Spearman correlation coefficients are color-coded to indicate positive (blue) or negative (red) associations. (**D**) LCK metagene gene expression scores in patients with endometrial cancer at different clinical stages. Data are presented as the mean ± SEM. *P < 0.05, ** *P* < 0.01, *** *P* < 0.001.

**Figure 2:**
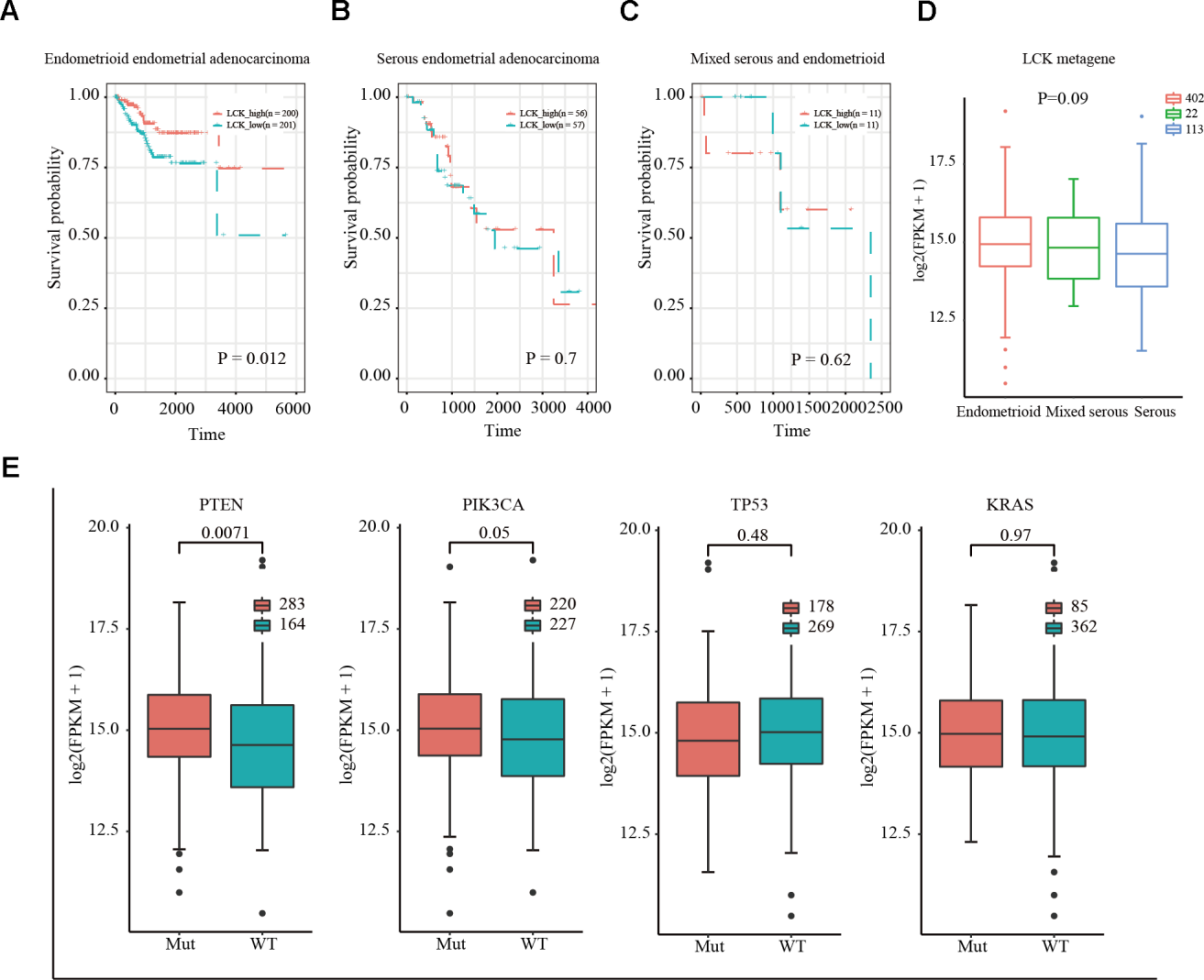
**Relationship between LCK metagene gene score and prognosis and gene mutation in endometrial cancer.** (**A**) Survival curves for endometrioid endometrial adenocarcinoma indicated that high expression of LCK metagene correlates with better clinical outcomes. (**B**) Survival curves for serous endometrial adenocarcinoma. (**C**) Survival curves for mixed serous and endometrioid. Data were analyzed in KM plotter. (**D**) LCK metagene scores of patients with different subtypes of endometrial cancer. (**E**) Somatic mutation data of PTEN, PIK3CA, TP53, and KRAS. Mut: mutant; WT: wild-type. Data are presented as the mean ± SEM. *P < 0.05, ** *P* < 0.01, *** *P* < 0.001.

Stromal cells, immune cells, and ESTIMATE scores were predicted by expression profile data using the ESTIMATE R package. Gene expression data were obtained from patients with different EC subtypes in TCGA database, and the correlation (cor) between the scores in patients and different immunoglobulin genes was calculated using the Spearman correlation coefficient (Figure 1A–1C). Functional annotation of the immune-system-related metagene clusters is presented in Supplementary Table 1. The endometrioid cohort in TCGA database is divided into three subtypes: endometrioid endometrial adenocarcinoma, serous endometrial adenocarcinoma, and mixed serous and endometrioid. In the three EC subtypes, except for the neoantigen score, the LCK metagene score showed a significant positive correlation with other types of immune-related scores: endometrioid endometrial adenocarcinoma (cor = 0.84), serous endometrial adenocarcinoma (cor = 0.83), and mixed serous and endometrioid (cor = 0.85). Next, we analyzed the distribution of the LCK metagene levels in three EC subtypes at different clinical stages of EC. The results revealed no significant differences in LCK metagene expression at different clinical stages (Figure 1D). Patients with each EC subtype were divided into two groups of high expression and low expression of LCK to analyze the prognosis of each group (Figure 2A–2C). We observed no significant differences in LCK metagene expression between the three subtypes in (Figure 2D). Furthermore, the prognostic analysis results showed that in the endometrioid endometrial adenocarcinoma subtype group, the survival rate of patients with high LCK metagene expression was markedly higher than that of the low expression group.

Next, we downloaded the somatic mutation data for *PTEN*, *PIK3CA*, *TP53*, and *KRAS*, which are commonly mutated genes in EC, and divided the patients into mutant and wild-type groups. The expression of LCK in the *PTEN*, *PIK3CA*, *TP53*, and *KRAS* groups and difference between the mutant and wild-type groups were assessed. The results showed that LCK metagene expression was higher in the *PTEN* and *PIK3CA* mutant groups than in the wild-type group, with no significant difference in LCK metagene expression between the *TP53* and *KRAS* mutant and wild-type groups (Figure 2E).

In summary, the LCK metagene is a representative genetic marker in the immune microenvironment of EC subtypes and can be used for prognostic evaluation of EC.

### Screening of representative genes in LCK metagene-related gene modules and identification of differentially expressed genes (DEGs) in high and low LCK metagene expression groups

**Figure 3:**
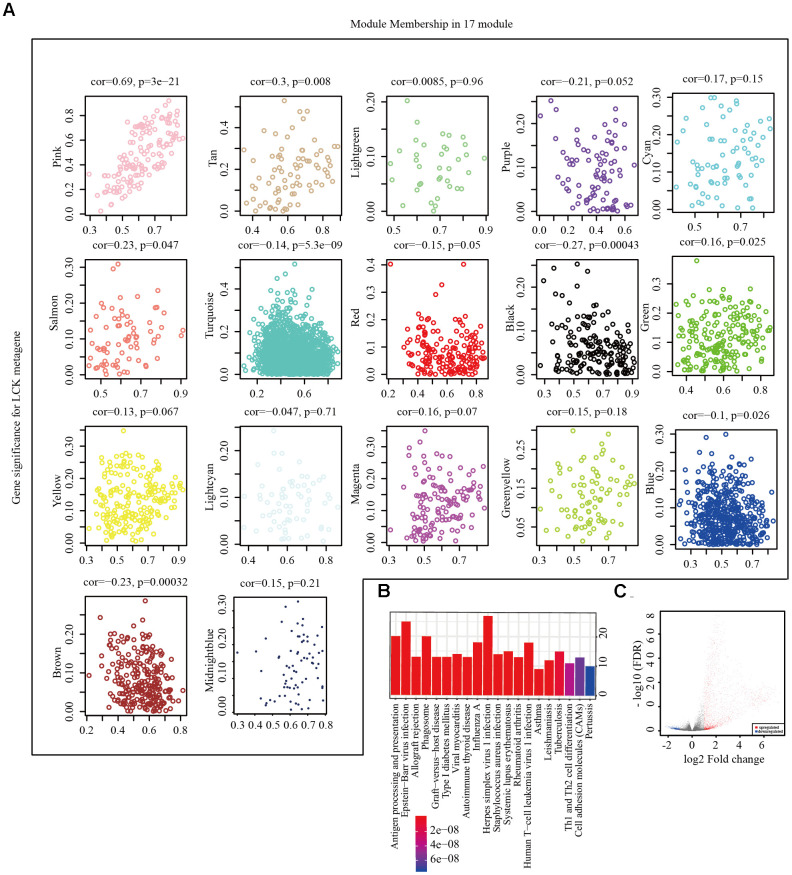
**Screening of representative genes in LCK metagene-related gene.** (**A**) Correlation between eigenvectors of 17 gene modules and LCK metagenes. (**B**) KEGG pathway enrichment analysis in pink module. (**C**) Volcano maps of DEGs. Red represents genes upregulated in patients with high LCK metagene scores, while blue represents genes downregulated in patients with low LCK metagene scores.

We next performed hierarchical clustering analysis (Supplementary Figure 1A), filtered out samples with distances of >120 as outliers, and obtained 546 samples. Weighted gene co-expression network analysis (WGCNA) was performed to construct a weighted co-expression network, and a β value of 6 was used to ensure a scale-free network (Supplementary Figure 1B, 1C). A total of 5000 genes were assigned to 19 co-expression modules (Supplementary Figure 1D). The number of genes corresponding to each module is shown in Supplementary Table 2. Two gene sets that could not be aggregated into other modules were excluded. We calculated the correlation between the feature vectors of the 17 modules and LCK metagene score (Figure 3A). The LCK metagene gene score was highly correlated with the pink module (R = 0.69). Next, we chose the pink (R = 0.69) module for Kyoto Encyclopedia of Genes and Genomes (KEGG) analysis. This module was enriched in 20 pathways related to various aspects of immunity, such as antigen processing and presentation, Th1 and Th2 cell differentiation, and cell adhesion molecules (Figure 3B). The limma-voom method was used to analyze the genetic differences between the high and low LCK expression groups, and 2,524 DEGs were obtained (Figure 3C). In the LCK high expression group, there were significantly more up-regulated genes than down-regulated genes.

### Exploration of prognostic markers related to the immune microenvironment of EC

**Figure 4:**
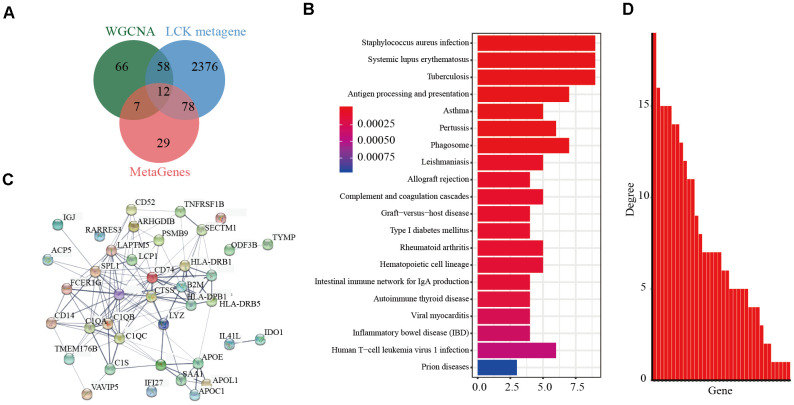
**Screening of prognostic markers related to the immune microenvironment of endometrial cancer.** (**A**) Veen diagram analysis showed co-expressed genes significantly associated with LCK metagene. (**B**) Gene KEGG pathway enrichment analysis showed 58 genes enriched in 20 pathways. The false discovery rate <0.05 as the threshold. (**C**) Protein interaction networks of these 58 genes. (**D**) The degree distribution of nodes in the network.

We then integrated the 141 genes from the pink modules of the LCK metagene and 2,524 DEGs between the high and low LCK metagene expression groups. Integration of the 70 selected genes (Supplementary Table 3), excluding 12 known immune-related metagenes, resulted in 58 genes (Figure 4A) (Supplementary Table 4). The R package clusterProfiler was used for KEGG enrichment analysis of these genes using a false discovery rate of <0.05 as the threshold (Figure 4B). Fifty-eight genes were enriched in 20 pathways, most of which were immune disease-related. The R package STRINGdb was used to analyze the protein network interaction of these 58 genes. After mapping these genes to the STRING database, a relationship network with 134 edges and 34 nodes was obtained (Figure 4C). Analysis of the distribution of nodes in the network (Figure 4D) showed that the connection degree of each node was very high (4.17, on average), indicating that these genes are closely related.

### Prognostic markers related to the immune microenvironment of EC

Next, we performed univariate cox survival analysis of the EC TCGA database to analyze the relationship between the expression of these 58 genes and patient prognosis. We subsequently included the clinical stage as a covariate in the analysis, with p-values < 0.05 as the threshold, to exclude its impact. A total of 11 genes met these conditions: *CD74*, *HLA-DRB5*, *CD52*, *HLA-DPB1*, *HLA-DRB1*, *TNFRSF1B*, *IGHA1*, *ODF3B*, *ACP5*, *LAPTM5*, and *IGLC2*. High expression of these genes was strongly correlated with prognosis, and we finally obtained 11 independent prognostic factors, as shown in Supplementary Table 5. The g: profiler was used to analyze the GO terms of these 11 genes. Four of these genes (*IGHA1*, *LAPTM5*, *ODF3B*, and *IGLC2*) were not enriched for any GO term and were eliminated. Finally, seven genes, *CD74*, *HLA-DRB5*, *CD52*, *HLA-DPB1*, *HLA -DRB1*, *TNFRSF1B*, and *ACP5*, were selected. The results showed 156 enriched GO terms associated with these 58 genes, most of which were related to immunity (Supplementary Table 6), including antigen processing, peptide or polysaccharide antigen presentation via MHC class II, regulation of T cell proliferation, and regulation of immune response-related cytokine production.

**Figure 5:**
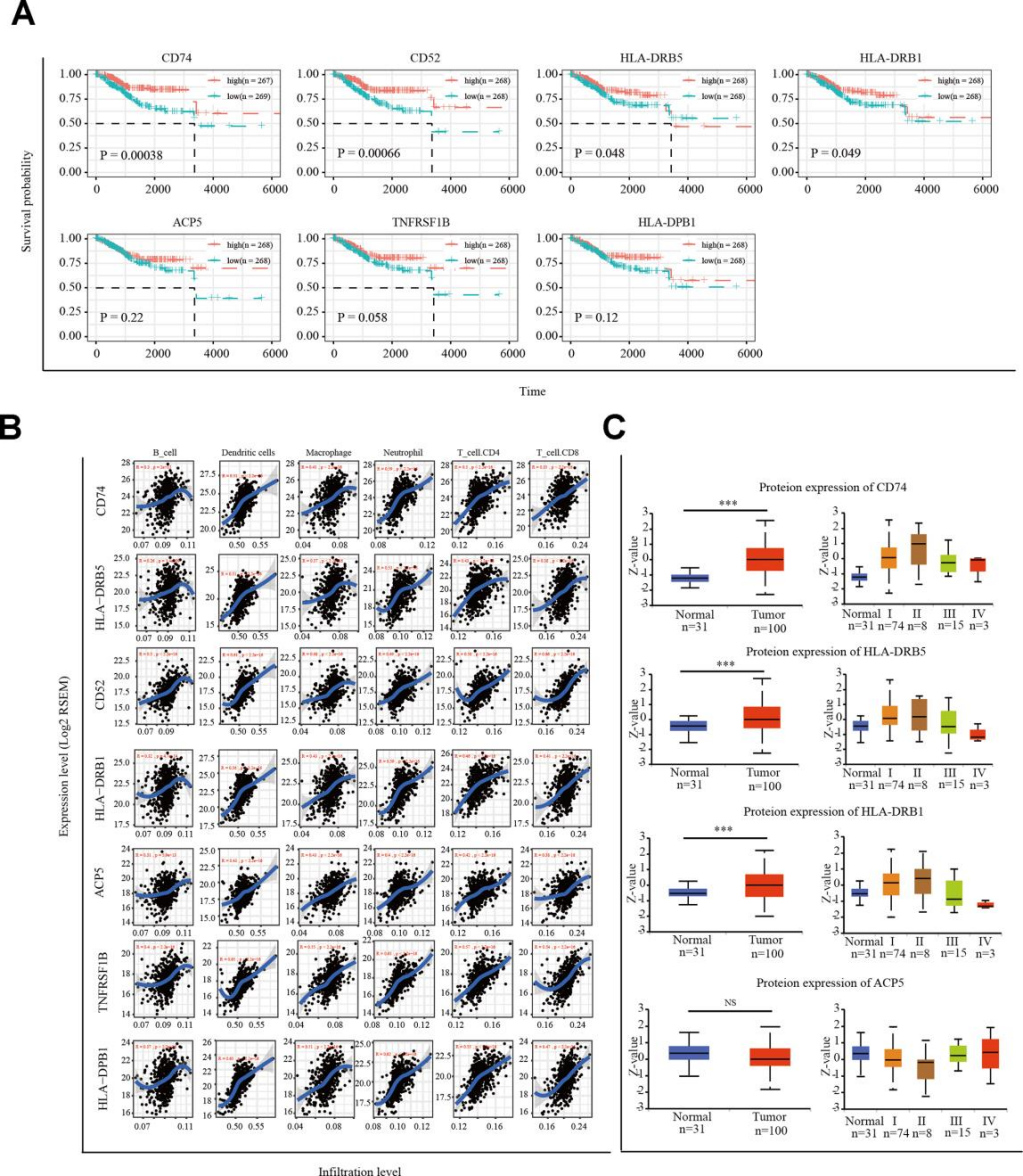
**Correlation of microenvironment related prognostic genes’ expression with immune infiltration level.** (**A**) Kaplan-Meier survival curve of 7 microenvironment related prognostic signature. (**B**) Immune cell infiltration analysis. A correlation coefficient of <0.3 indicates no correlation and a value of >0.3 indicates a positive correlation. (**C**) UALCAN website analysis CD74, HLA-DRB5, HLA-DRB1 and ACP5 protein expression. *P < 0.05, ** *P* < 0.01, *** *P* < 0.001.

Samples from patients with EC in TCGA were divided into two groups according to the median expression levels of the seven prognosis-related genes of the EC immune microenvironment. Prognostic differences between the high and low expression groups of these seven genes were analyzed. The results showed that patients with high expression of these seven genes had higher survival rates (Figure 5A).

Next, we used the tumor immune estimation resource (TIMER) algorithm to analyze six infiltrating-immune cells (CD4^+^ T cells, CD8^+^ T cells, B cells, neutrophils, macrophages, and dendritic cells) in the uterus and the correlation between the expression of the seven selected genes and level of immune infiltration. The results showed that the expression of *CD74*, *HLA-DRB5*, *CD52*, *HLA-DPB1*, *HLA-DRB1*, *TNFRSF1B*, and *ACP5* was significantly positively correlated with the level of immune infiltration (Figure 5B). We analyzed the protein expression of these seven genes in EC tissues using the online tool UALCAN (http://ualcan.path.uab.edu/index.html). Available data on the UALCAN platform showed revealed the CD74, HLA-DRB5, HLA-DRB1, and ACP5 protein levels in EC tissues and normal endometrial tissues (Figure 5C). CD52 and TNFRSF1B protein expression was not predicted.

### Specimen verification

**Figure 6:**
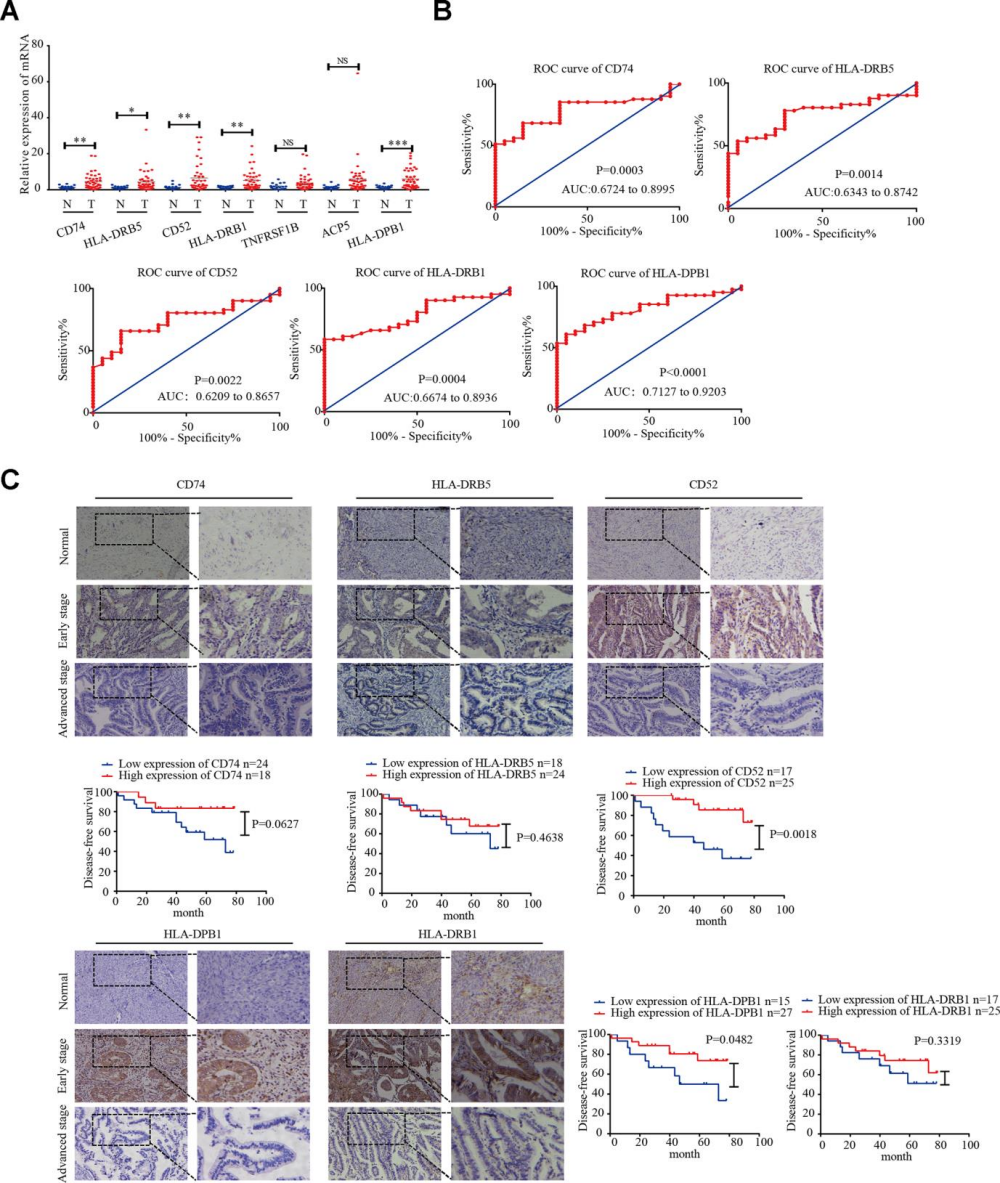
**CD74, HLA-DRB5, CD52, HLA-DPB1, and HLA-DRB1 in the microenvironment of related prognostic markers in endometrial cancer.** (**A**) Expression of *CD74*, *HLA-DRB5*, *CD52*, *HLA-DPB1*, *HLA-DRB1*, *TNFRSF1B*, and *ACP5* in 41 endometrial cancer tissues and 20 normal tissues was determined by qRT-PCR. (**B**) ROC curve of 5 msicroenvironment-related prognostic signature. (**C**) Expression of CD74, HLA-DRB5, CD52, HLA-DPB1 and HLA-DRB1 was detected by immunohistochemistry in endometrial cancer (n = 42) and normal endometrial tissue (n = 20). Disease-free survival curves for CD74, HLA-DRB5, CD52, HLA-DPB1 and HLA-DRB1 in 42 endometrial carcinoma cases. *P < 0.05, **P < 0.01, ***P < 0.001.

Next, we detected the mRNA expression of *CD74*, *HLA-DRB5*, *CD52*, *HLA-DPB1*, *HLA-DRB1*, *TNFRSF1B*, and *ACP5* in 41 EC tissues and 20 normal endometrial tissues by real-time PCR. The results showed that the expression levels of *CD74*, *HLA-DRB5*, *CD52*, *HLA-DPB1*, *HLA-DRB1* were higher in EC tissues than in normal endometrial tissues (Figure 6A). Receiver operating characteristic (ROC) curve analysis was performed, and the correlation area under the curve was used to confirm the diagnostic efficacy of the gene expression levels (Figure 6B). The results suggest that *CD74*, *HLA-DRB5*, *CD52*, *HLA-DPB1* and *HLA-DRB1* expression levels can discriminate between EC and normal endometrial tissue. Furthermore, CD74, HLA-DRB5, CD52, HLA-DPB1 and HLA-DRB1 protein expression levels were detected by immunohistochemistry in 42 EC tissues and 20 normal endometrial tissues. The results showed that the high expression rate of CD74 protein expression in early-stage EC was 54.5%, which was higher than that of in normal endometrial tissue (20%, P = 0.0289). The high expression rate of HLA-DRB5 protein expression in early-stage EC was 59.1%, which was higher than that of in normal endometrial tissue (25%, P = 0.0334). The high expression rate of CD52 protein expression in early-stage EC was 63.6%, which was higher than that of in normal endometrial tissue (25%, P = 0.0157). The high expression rate of HLA-DPB1 protein expression in early-stage EC was 54.5%, which was higher than that of in normal endometrial tissue (20%, P = 0.0068). The high expression rate of HLA-DRB1 protein expression in early-stage EC was 72.2%, which was higher than that of in normal endometrial tissue (40%, P = 0.0124). These five proteins were highly expressed in early-stage EC tissues compared to in normal endometrial tissue. In advanced EC tissues, there was not significant difference in the high expression rate of these five proteins compared to in normal tissues. The disease-free survival curves indicated that high expression of CD52 and HLA-DPB1 is correlated with high survival rates in EC (Figure 6C).

## DISCUSSION

Abundant infiltrating-immune cells and cytokines are typically observed in EC tissues, which can stimulate the endogenous anti-tumor immune response [24], indicating that patients with EC may benefit from immunotherapy. Exploring genes related to the EC immune environment that can predict prognosis is a pivotal step for finding treatment targets for immunotherapy.

In this study, we first assessed the correlation between different types of EC and different immune-related scores by analyzing different EC subtypes in TCGA database: endometrioid endometrial adenocarcinoma, serous endometrial adenocarcinoma, and mixed serous and endometrioid. The results showed that the LCK metagene score was the highest relative to other types of immune-related scores. Studies have shown that in breast cancer, the LCK metagene has a high co-expression level with immune characteristics and is significantly positively correlated with the histological TIL count. This single representative measure of immune infiltration is correlated with global genomic metrics. In one study, microarray analysis of 1,781 primary breast cancer samples in 12 data sets was performed to determine the correlation between immune system-related metagenes and clinical parameters and survival rates. A large cluster of nearly 600 genes with functions in immune cells was consistently obtained in all datasets, among them, the LCK metagene showed very high immune prognostic value. In ER-negative and HER2 overexpression ER-positive EC, patients with high expression of LCK had a better prognosis [25–26]. In EC, no metagene has been reported to be related to the immune microenvironment. Based on the prominent role of the LCK metagene in breast cancer, it was used as a research target in EC. Our results showed that the LCK metagene is strongly correlated with the immune microenvironment of EC. The LCK metagene consists of 47 genes (*ARHGAP15*, *ARHGAP25*, *CCL5*, *CCR2*, *CCR7*, *CD2*, *CD247*, *CD27*, *CD3D*, *CD48*, *CD53*, *CORO1A*, *CSF2RB*, *EVI2B*, *FGL2*, *GIMAP4*, *GIMAP5*, *GMFG*, *GZMA*, *HC1*, *GZM*, *IL2RG*, *IL7R*, *INPP5D*, *IRF8*, *ITK*, *KLRK1*, *LCK*, *LCP2*, *LPXN*, *LTB*, *PIK3CD*, *PLAC8*, *PRG1*, *PRKCB1*, *PTPRC*, *RAC2*, *SAMSSN1*, *SCYA5*, *SELL*, *SD2D1A*, *SLA*, *SLAMF1*), which are all directly or indirectly involved in T cell-mediated immunity. For example, CCL5 is a chemotactic agent for memory T helper cells and eosinophils [27–28]. Moreover, the protein encoded by *CD27* is a member of the TNF receptor superfamily. This receptor is necessary for the generation and long-term maintenance of T cell immunity. It binds to ligand CD70 and plays a key role in regulating B cell activation and immunoglobulin synthesis [29]. LCK is a cytoplasmic tyrosine kinase of the Src family expressed in T cells and natural killer cells. It is relatively specific in lymphocytes, particularly in mature resting T lymphocytes, activating signal transduction in T cells and playing an essential regulatory role of differentiation. LCK activation is a core step in T cell activation. Before this step, LCK forms a non-covalent bond with the CD4 and CD8 complex receptors via cysteine at the N-terminus [30]. Therefore, selective inhibition of LCK can be used to treat T cell-mediated autoimmune diseases, inflammatory diseases, and organ transplant rejection [31–32]. Stimulating an abnormal LCK signal to enhance the reset of the PD-1 blockade has become a new targeted molecular approach for cancer treatment [33].

TCGA database analysis showed that in the endometrioid endometrial adenocarcinoma subtype, the prognosis of patients with high LCK expression was significantly better than that of the low expression group. These results suggest that the LCK metagene is a prognostic marker in EC. The most common mutant genes of EC were *PTEN*, *PIK3CA*, *TP53*, and *KRAS* [34]. We downloaded the somatic mutation data of these four genes and divided the patients into mutant and wild-type groups. The LCK metagene expression of each group was analyzed. Among them, expression of the LCK metagene was significantly increased in the *PTEN* and *PIK3CA* mutant groups. *PTEN* mutation or deletion is one of the most significant molecular characteristics of EC. The mutation rates in low- and high-grade endometrioid carcinomas are 67.0% and 90.0%, respectively, and 2.7% in serous carcinomas [35]. The oncogene *PIK3CA* has a mutation rate of 52% in type I EC and 33% in type II EC [36–37]. Compared with *PTEN* mutations that occur in the early stages of the lesion, *PIK3CA* mutations tend to occur in the middle and late stages of disease. Furthermore, *PIK3R1* mutations destabilize PTEN, which is a key event leading to tumor development [38]. Therefore, as a representative metagene in the immune microenvironment of EC, the LCK metagene is a potential research target.

Next, we used the LCK metagene members as the core object and performed WGCNA to detect representative genes from LCK-related gene modules and constructed a weighted co-expression network. We also analyzed the DEGs between samples with high and low LCK metagene scores to identify co-expressed genes whose mRNA levels were significantly correlated with LCK metagenes. By evaluating the overlap between co-expressed genes significantly related to LCK and exploring the functions of these genes through enrichment analysis, we found multiple enriched immune-related GO terms, particularly the T cell receptor signaling pathway and T cell activation. Survival analysis and prediction revealed seven potential immune-related diagnostic and prognostic markers. These included *CD74*, *HLA-DRB5*, *CD52*, *HLA-DPB1*, *HLA-DRB1*, *TNFRSF1B*, and *ACP5*. We used the TIMER algorithm to calculate the correlation between the expression of these seven genes and degree of infiltration of CD4^+^ T cells, CD8^+^ T cells, B cells, neutrophils, macrophages, and dendritic cells. All genes were significantly positively correlated with cellular infiltration.

HLA is a highly genetically polymorphic group of genes that is the main component of specific immune recognition and the immune response in the body [39]. HLA complexes are composed of many genes and can be approximately divided into three categories: class I and class II molecules are the main types involved in antigen presentation and related immune responses. HLA-I includes HLA-A, HLA-B, and HLA-C; HLA-II includes HLA-DR, HLA-DQ, and HLA-DP. After MHC-I binds to the peptide, it is presented on the cell surface for recognition by CD8^+^ T cells; HLA-II molecules bind to CD4 on CD4^+^ T cells and help the T cell antigen receptor transmit activation signals to T cells to promote their activation. Eradication of tumors by the immune system depends on the effective activation of T cell responses [40]. Studies have shown that the high expression level of MHC-II molecules in hepatocellular carcinoma tissues is an effective prognostic marker of prolonged relapse-free survival time in liver cancer [41]. Baccar et al. performed HLA-II staining of 80 surgically resected breast malignant and non-malignant tissue sections. The results showed that CD99 (+) HLA-II (-) was the worst prognostic marker [42]. These findings suggest that in EC, HLA-DRB1 and HLA-DRB5 are new markers for the prognosis of patients and provide new targets for targeted therapy through T cell activation.

CD74, the constant chain of MHC-II, can assist it in reaching the acidic endosome compartment for intracellular antigen processing, participate in MHC-II-mediated antigen presentation, and play an important role in the occurrence and development of tumors [43]. For many years, studies of cancer immunotherapy focused on cytotoxic CD8 T cells. However, stimulation of CD4 helper T cells is essential for promoting and maintaining immune memory. Therefore, a good therapeutic target should cause a two-dimensional T cell response. CD74 is necessary for the MHC class II heterodimer to correctly guide cells to load peptides and be expressed on the surface of antigen-presenting cells. Mensali et al. showed that CD74-expressing dendritic cells can prime CD4 and CD8 T cells from a naïve population [44]. In EC tissues, positive CD4 and CD8 are good prognostic markers, and PD-L1 and CD4^+^ helper T cells may be suitable targets for improving the survival rate by enhancing chemical sensitivity [45]. In brain metastatic tumor cells, the highly expressed CD74 promotes the normal processing mechanism of HLA-II and binding of complex HLA peptides, which is crucial for improving the prognosis of patients [46]. These studies provide useful information showing that CD74 can be used as a treatment strategy to incorporate immunotherapy into EC. CD52 can be expressed on the cell membrane surface of B-lymphocytic tumors, and targeted therapy using anti-CD52 monoclonal antibodies has attracted increased attention at home and abroad. This single drug or combined chemotherapy can benefit some patients with refractory and relapsed CLL [47].

Then, we analyzed the diagnostic efficacy and prognostic value of *CD74*, *HLA-DRB5*, *CD52*, *HLA-DPB1*, *HLA-DRB1*, *TNFRSF1B*, and *ACP5* in EC. We detected the expression of these seven genes in EC and normal endometrial tissue by real-time PCR. The results showed that *CD74*, *HLA-DRB5*, *CD52*, *HLA-DPB1*, and *HLA-DRB1* were significantly overexpressed in EC tissues. In EC, the functions of CD74, HLA-DRB5, CD52, HLA-DPB1, and HLA-DRB1 are poorly understood. Moreover, the ROC results showed that the expression levels of these genes can distinguish EC from normal endometrial tissue. Immunohistochemical analysis revealed that CD74, HLA-DRB5, CD52, HLA-DPB1, and HLA-DRB1 were highly expressed in early-stage EC tissues compared to in normal endometrial tissue. In advanced EC tissues, there was no significant difference in the high expression rate of these five proteins compared to in normal tissues. Survival analysis showed that patients highly expressing CD52 and HLA-DPB1 had longer disease-free survival. The expression of CD74, HLA-DRB5, and HLA-DRB1 in the high and low expression groups was not significantly related to survival rates. Combined with the survival curve of the prognostic signature from TCGA dataset shown in Figure 5A and immune cell infiltration analysis shown in Figure 5B, in EC, CD74, HLA-DRB5, and HLA-DRB1 are potential microenvironment-related prognosis factors. The EC microenvironment is very complex, the composition of which and its correlation with EC prognosis remain poorly understood compared to other malignancies. Thus, studies of larger sample sizes and involving *in vitro* experiments are needed. Our results provide new prognostic assessment and targets for drug therapy for patients with EC and can guide individualized cancer immunotherapies.

In this research, using ESTIMATE algorithm-based immune scoring and TCGA EC cohort information analysis, immune-related genes of EC were screened and prognostic characteristics were established. Notably, through specimen verification, we found that the CD74, HLA-DRB5, CD52, HLA-DPB1 and HLA-DRB1 proteins are high expressed in early-stage EC tissues. Patients with high expression of CD52 and HLA-DPB1 show better prognosis. In summary, our research provides targets in the immune microenvironment for the molecular therapy of EC.

## MATERIALS AND METHODS

### Data sources and pre-processing

TCGA-UCEC standardized FPKM data (https://bioinformatics.mdanderson.org/), somatic mutation, and clinical information were downloaded from the UCSC Xena official website (https://xena.ucsc.edu/). The 13 types of immune metagenes and immune function data were downloaded from DOI: 10.1158/0008-5472. CAN-16-3478. The scores of 13 types of immune metagenes were obtained by calculating the average value of log2 (FPKM + 1). Six types of immune cell scores (B_cell, CD4_Tcell, CD8_Tcell, neutrophil, macrophage, dendritic) were obtained from mRNA expression data using the TIMER package (https://cistrome.shinyapps.io/timer/). The StromalScore and ImmuneScore were calculated using the ESTIMATE R package (https://bioinformatics.mdanderson.org/estimate/index.html). ESTIMATE R package uses expression profile data to predict the scores of stromal cells and immune cells, and then predicts the content of these two cells; ImmuneSore: immune cell score, StromalScore: stromal cell score ESTIMATEScore: comprehensive score. The expression of the CD74, HLA-DRB5, HLA-DRB1, and ACP5 proteins in EC tissues was analyzed using the online tool UALCAN (http://ualcan.path.uab.edu/index.html).

### Screening representative genes in the EC immune microenvironment

The Spearman correlation coefficient was used to calculate the correlation between different immune-related scores in different EC subtypes. The results showed that the LCK metagene score was the highest relative to other types of immune-related scores. Among the three subtypes (endometrioid endometrial adenocarcinoma: 0.84, serous endometrial adenocarcinoma: 0.83, mixed serous and endometrioid: 0.85), LCK metagene was selected as representative of the EC immune microenvironment gene. The samples were divided into two groups according to the median of LCK metagene mRNA expression level, and the Kaplan-Meier (KM) survival curves of the two groups were drawn. We also analyzed the relationship between the LCK metagene mRNA expression level and *PTEN, PTK3CA, TP53,* and *KRAS* mutations.

### Analysis of LCK metagene-related modules by WGCNA

Screening for genes with a median absolute deviation of the top 75% and genes with a MAD greater than 0.01 was performed using the WGCNA R package to construct a gene co-expression network with genetic methods used to generate a dynamic shear module and for cluster analysis of the module. Genes with similar expression levels were divided into the same module; the important parameters were as follows: minModuleSize = 30, merge CutHeight = 0.25. The results showed that the 141 genes in the pink module were highly correlated with the LCK metagene (cor = 0.69). The clusterProfiler R package was used for KEGG analysis of this module (FDR < 0.05).

### Screening immune microenvironment genes related to prognosis

According to the LCK metagene score, the samples were divided into two groups of LCK high and low, and the differential expression analysis of genes was performed using the limma R package (FDR < 0.1, |log_2_ (fold-change)| > 1), and KEGG analysis was performed on the 58 genes of the intersection. In order to identify genes with prognostic value in the immune microenvironment, we performed univariate cox survival analysis and used the survminer R package to draw KM survival curves.

### Patients and samples

EC and normal endometrial tissues were collected during surgical treatment at Shengjing Hospital Affiliated to China Medical University from 2011 to 2017. Fresh tissues included 41 EC tissues and 20 normal endometrial tissues, which were evaluated by PCR. There were 66 paraffin-embedded specimens, including 42 EC tissues and 20 normal endometrial tissues, with 22 cases of early EC and 20 cases of advanced EC. The inclusion criteria were as follows: 1. The patient had never been administered radiochemotherapy and other anti-tumor treatment before specimen collection. 2. The patient had no history of other gynecological malignancies or metabolic and infectious diseases. Under sterile conditions, fresh tissue samples were obtained and stored at -80°C. All pathological diagnoses were verified by two pathologists. The clinical data of all enrolled patients were collected and counted, including patient age, pathological type, FIGO stage, degree of differentiation, muscular layer infiltration, and lymph node metastasis, and informed consent was obtained from all subjects. This study was approved by the Ethics and Ethics Committee of Shengjing Hospital Affiliated to China Medical University.

### RNA extraction and quantitative RT-PCR

RNA was extracted from tissues and cells using TRIzol (Takara, Shiga, Japan), According to the instructions of the PrimeScript™ RT reagent Kit with gDNA Eraser (Takara), reverse transcription was performed. qRT-PCR was conducted according to the instructions of the SYBR® TB Green™ Premix Ex Taq II (Takara). PCR-specific primers were designed by Sangon Biotech Co., Ltd. (Shanghai, China). The fold-change in expression was calculated using the 2^-ΔΔCt^ method, with GAPDH used as an internal control. The primer sequences are listed in Supplementary Table 7.

### Immunohistochemistry

Histopathological specimens were fixed in formalin and embedded in paraffin, and 5-μm serial sections were prepared. After antigen repair operation, immunohistochemistry analysis was performed using a kit (ZSGB-BIO, Beijing, China). After saturating the endogenous peroxidase activity with 3% H_2_O_2_ and blocking with goat serum, the prepared antibody was added dropwise and incubated overnight at 4°C. The primary antibodies information are listed in Supplementary Material 8: Supplementary Table 8. On the next day, the samples were washed with PBS. After horseradish peroxidase-conjugated secondary antibody was added dropwise, DAB color development and hematoxylin counterstaining were performed and the samples were evaluated by microscopy. The scoring method is based on whether the cell cytoplasm has a brownish yellow or brown color as a positive result. No staining, 0; light yellow, 1; yellow, 2; and brown and sepia, 3. According to the percentage of positive cells, the mean value after scoring, with negative count as 0, the scores were assigned as follows: percentage of positive cells less than 10%, 1; ≥10–50%, 2; >50–75%, 3; and ≥75%, 4. The product of 2 scores was considered as the total score, and the results were interpreted as follows: ≤2, negative; 3–4, weak positive (+); 5–8, medium positive (++); and 9–12, strong positive (+++). Low expression was indicated by -/+, and high expression was indicated by ++/+++, respectively. The results were evaluated by two senior pathologists who were blinded to the patients’ data, and each slice was independently observed to determine the positive cell count and evaluate the background. In cases of disagreement, a third pathologist made the judgement.

### Statistical analysis

GraphPad Prism 8 software (GraphPad, Inc., La Jolla, CA, USA) was used for statistical analysis of the experimental data. All data are expressed as the mean ± SEM. Student’s *t*-test was used to compare differences between the two groups of samples. Survival curves were plotted using the results of Kaplan-Meier (KM) analysis, and disease-free survival was defined as the time from the date of diagnosis to the time of progression/death or last follow-up. *P* < 0.05 was defined as statistically significant.

### Ethics approval

The study protocol was reviewed and approved by the Scientific Research and New Technology Ethical Committee of the Shengjing Hospital of China Medical University. Ethical number: 2018PS251K.

## Supplementary Material

Supplementary Figure 1

Supplementary Tables 1, 2, 3, 4 and 5

Supplementary Table 6

Supplementary Tables 7 and 8
