# Supplementary material for: Identification of novel prognosis-related genes in the endometrial cancer immune microenvironment
Source: Aging (Albany NY). 2020 Nov 6;12(21):22152–73. doi: 10.18632/aging.104083 (PMC7695382; doi:10.18632/aging.104083)
Supplement: Supplementary Figure 1 [file aging-12-104083-s001..pdf]

SUPPLEMENTARY FIGURE

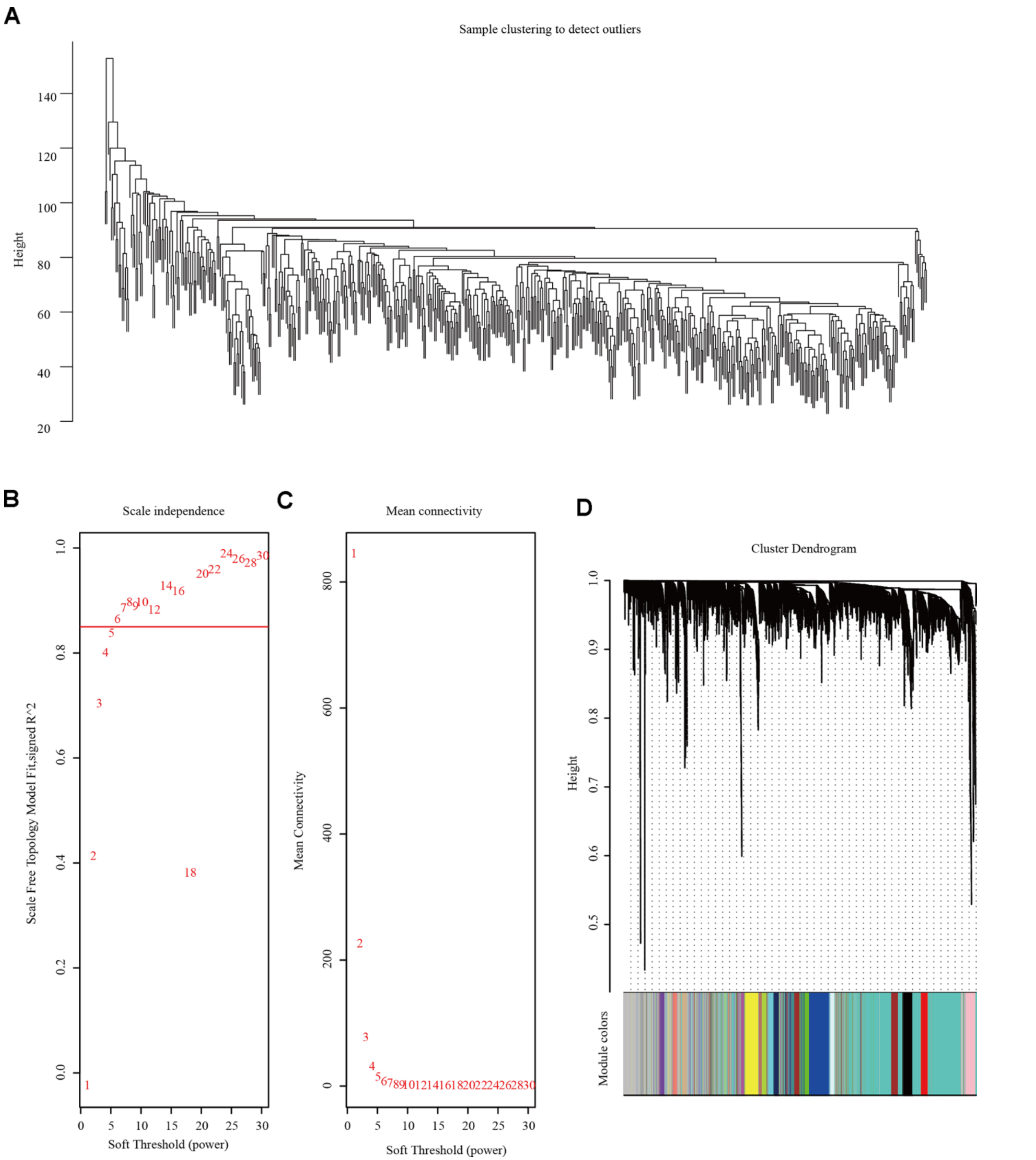

Supplementary Figure 1. LCK metagenes-related gene modules mined through WGCNA. (**A**) Sample clustering analysis. (**B–C**) Analysis of network topology under various soft-thresholding powers. (**D**) Gene dendrogram and module colors.
