# Supplementary material for: Identification of novel prognosis-related genes in the endometrial cancer immune microenvironment
Source: Aging (Albany NY). 2020 Nov 6;12(21):22152–73. doi: 10.18632/aging.104083 (PMC7695382; doi:10.18632/aging.104083)
Supplement: Supplementary Tables 1, 2, 3, 4 and 5 [file aging-12-104083-s002..pdf]

SUPPLEMENTARY TABLES

Supplementary Table 1. Functional annotation of the immune-system-related metagene clusters.

Metagene	Incorporated genes
LCK	Genes in this cluster contain ARHGAP15 ARHGAP25 CCL5 CCR2 CCR7 CD2 CD247 CD27 CD3D CD48 CD53 CORO1A CSF2RB EVI2B FGL2 GIMAP4 GIMAP5 GMFG GZMA GZMK HCLS1 IL10RA IL2RG IL7R INPP5D IRF8 ITK KLRK1 LCK LCP2 LPXN LTB PIK3CD PLAC8 PRG1 PRKCB1 PTPRC RAC2 SAMSSN1 SCYA5 SELL SD2D1A SLA SLAMF1 STAT4 TNFRSF7 TRBC1
Tfh	This cluster contain CD200 CXCL13 FBLN7 ICOS SGPP2 SH2D1A TIGIT PDCD1
Tregs	This cluster contain FOXP3 C15orf53 IL5 CTLA4 IL32 GPR15 IL4
Cytolytic	This cluster contain GZMA PRF1
MHC2	This cluster contain HLA-DMA HLA-DQB1 HLA-DRA HLA-DRB4
NK	This cluster contain KLRF1 KLRC1
Macrophages	This cluster contain FUCA1 MMP9 LGMN HS3ST2 TM4SF19 CLEC5A GPNMB C11orf45 CD68 CYBB
MHC1	This cluster contain HLA-A HLA-B HLA-C HLA-F HLA-G HLA-J
STAT1	This cluster contain CXCL10 CXCL11 GBP1 STAT1
IF_I	This cluster contain DDX58 HERC6 IFI44 IFI44L IFIT1 IFIT2 MX1 OAS1 OAS3 RSAD2
Co_stimulation	This cluster contain CD2 CD226 CD27 CD28 CD40 CD40LG CD58 CD70 ICOS ICOSLG SLAMF1 TNFRSF18 TNFRSF25 TNFRSF4 TNFRSF8 TNFRSF9 TNFSF14 TNFSF15 TNFSF18 TNFSF4 TNFSF8 TNFSF9
Co_inhibition	This cluster contain BTLA C10orf54 CD160 CD244 CD274 CTLA4 HAVCR2 LAG3 LAIR1 LGALS9 PDCD1LG2 PVRL3 TIGIT

Supplementary Table 2. The number of genes corresponding to 19 module.

Module	Number
Black	166
Blue	498
Brown	241
Cyan	74
Green	196
Greenyellow	83
Grey	912
Grey-60	52
Lightcyan	66
Lightgreen	37
Magenta	129
Midnightblue	73
**Pink**	**141**
Purple	86
Red	171
Salmon	75
Tan	77
Turquoise	1724
Yellow	199

Supplementary Table 3. The integration of the 70 selected genes ID.

ID
ENSG00000008517
ENSG00000011600
ENSG00000019582
ENSG00000025708
ENSG00000028137
ENSG00000066336
ENSG00000090382
ENSG00000100342
ENSG00000100985
ENSG00000102575
ENSG00000104951
ENSG00000106565
ENSG00000111348
ENSG00000122862
ENSG00000125347
ENSG00000125730
ENSG00000128340
ENSG00000130203
ENSG00000130208
ENSG00000131203
ENSG00000132465
ENSG00000133321
ENSG00000136167
ENSG00000141574
ENSG00000143119
ENSG00000158869
ENSG00000159189
ENSG00000162511
ENSG00000163131
ENSG00000165949
ENSG00000166710
ENSG00000168899
ENSG00000169245
ENSG00000169442
ENSG00000170458
ENSG00000173369
ENSG00000173372
ENSG00000173432
ENSG00000177989
ENSG00000179344
ENSG00000182326
ENSG00000196126
ENSG00000198502

ENSG00000204257
ENSG00000204287
ENSG00000204642
ENSG00000211592
ENSG00000211598
ENSG00000211644
ENSG00000211653
ENSG00000211666
ENSG00000211677
ENSG00000211679
ENSG00000211890
ENSG00000211892
ENSG00000211893
ENSG00000211895
ENSG00000211896
ENSG00000211897
ENSG00000211899
ENSG00000211949
ENSG00000223865
ENSG00000229391
ENSG00000231389
ENSG00000234745
ENSG00000239951
ENSG00000240065
ENSG00000241351
ENSG00000243466
ENSG00000271503

Supplementary Table 4. The 58 Common genes ID.

ID
ENSG00000019582
ENSG00000166710
ENSG00000196126
ENSG00000211895
ENSG00000130203
ENSG00000211592
ENSG00000211677
ENSG00000165949
ENSG00000211896
ENSG00000125730
ENSG00000159189
ENSG00000198502
ENSG00000100342
ENSG00000173372
ENSG00000223865
ENSG00000173369
ENSG00000133321
ENSG00000011600
ENSG00000111348

ENSG00000162511
ENSG00000211679
ENSG00000170458
ENSG00000211893
ENSG00000122862
ENSG00000211890
ENSG00000211899
ENSG00000158869
ENSG00000211897
ENSG00000231389
ENSG00000106565
ENSG00000168899
ENSG00000130208
ENSG00000239951
ENSG00000025708
ENSG00000104951
ENSG00000182326
ENSG00000163131
ENSG00000102575
ENSG00000240065
ENSG00000211892
ENSG00000136167
ENSG00000211598
ENSG00000243466
ENSG00000211666
ENSG00000211653
ENSG00000229391
ENSG00000090382
ENSG00000131203
ENSG00000132465
ENSG00000211644
ENSG00000169442
ENSG00000173432
ENSG00000211949
ENSG00000241351
ENSG00000177989
ENSG00000066336
ENSG00000028137
ENSG00000141574

Supplementary Table 5. Genes with prognostic value.

Genes	Symbol	HR	pvalue	Low 95% CI	High 95%CI
ENSG00000019582	CD74	0.783657	0.00074	0.680156	0.902907
ENSG00000198502	HLA-DRB5	0.83873	0.00110	0.754685	0.932135
ENSG00000169442	CD52	0.815082	0.00498	0.706682	0.94011
ENSG00000223865	HLA-DPB1	0.82427	0.00795	0.714643	0.950714
ENSG00000196126	HLA-DRB1	0.84914	0.01448	0.744817	0.968075
ENSG00000028137	TNFRSF1B	0.790104	0.01906	0.648852	0.962106
ENSG00000211895	IGHA1	0.91981	0.02056	0.856994	0.987231
ENSG00000177989	ODF3B	0.880537	0.03447	0.782588	0.990745
ENSG00000102575	ACP5	0.845597	0.03732	0.722112	0.990198
ENSG00000162511	LAPTM5	0.83494	0.03744	0.704476	0.989566
ENSG00000211677	IGLC2	0.931208	0.04685	0.868008	0.999009

HR: hazard ratio; CI: confidence interval
