# Supplementary material for: Identification of novel prognosis-related genes in the endometrial cancer immune microenvironment
Source: Aging (Albany NY). 2020 Nov 6;12(21):22152–73. doi: 10.18632/aging.104083 (PMC7695382; doi:10.18632/aging.104083)
Supplement: Supplementary Table 6 [file aging-12-104083-s003..docx]

**Supplementary Table 6: GO enrichment of 7 immune-related genes**

| **ID** | **Description** | ***P* value** | **Gene** |
| --- | --- | --- | --- |
| GO:0019886 | antigen processing and presentation of exogenous peptide antigen via MHC class II | 4.92E-08 | CD74;HLA-DRB5;HLA-DPB1;HLA-DRB1 |
| GO:0002495 | antigen processing and presentation of peptide antigen via MHC class II | 5.55E-08 | CD74;HLA-DRB5;HLA-DPB1;HLA-DRB1 |
| GO:0002504 | antigen processing and presentation of peptide or polysaccharide antigen via MHC class II | 5.78E-08 | CD74;HLA-DRB5;HLA-DPB1;HLA-DRB1 |
| GO:0002478 | antigen processing and presentation of exogenous peptide antigen | 5.07E-07 | CD74;HLA-DRB5;HLA-DPB1;HLA-DRB1 |
| GO:0019884 | antigen processing and presentation of exogenous antigen | 5.93E-07 | CD74;HLA-DRB5;HLA-DPB1;HLA-DRB1 |
| GO:0048002 | antigen processing and presentation of peptide antigen | 6.90E-07 | CD74;HLA-DRB5;HLA-DPB1;HLA-DRB1 |
| GO:0019882 | antigen processing and presentation | 1.41E-06 | CD74;HLA-DRB5;HLA-DPB1;HLA-DRB1 |
| GO:0060333 | interferon-gamma-mediated signaling pathway | 6.16E-06 | HLA-DRB5;HLA-DPB1;HLA-DRB1 |
| GO:0071346 | cellular response to interferon-gamma | 4.76E-05 | HLA-DRB5;HLA-DPB1;HLA-DRB1 |
| GO:0034341 | response to interferon-gamma | 6.42E-05 | HLA-DRB5;HLA-DPB1;HLA-DRB1 |
| GO:0050852 | T cell receptor signaling pathway | 6.71E-05 | HLA-DRB5;HLA-DPB1;HLA-DRB1 |
| GO:0050670 | regulation of lymphocyte proliferation | 7.32E-05 | CD74;HLA-DPB1;TNFRSF1B |
| GO:0032944 | regulation of mononuclear cell proliferation | 7.43E-05 | CD74;HLA-DPB1;TNFRSF1B |
| GO:0070663 | regulation of leukocyte proliferation | 8.89E-05 | CD74;HLA-DPB1;TNFRSF1B |
| GO:0046651 | lymphocyte proliferation | 0.000162 | CD74;HLA-DPB1;TNFRSF1B |
| GO:0032943 | mononuclear cell proliferation | 0.000166 | CD74;HLA-DPB1;TNFRSF1B |
| GO:0070661 | leukocyte proliferation | 0.000212 | CD74;HLA-DPB1;TNFRSF1B |
| GO:0050863 | regulation of T cell activation | 0.000248 | CD74;HLA-DPB1;TNFRSF1B |
| GO:0050851 | antigen receptor-mediated signaling pathway | 0.000253 | HLA-DRB5;HLA-DPB1;HLA-DRB1 |
| GO:0002718 | regulation of cytokine production involved in immune response | 0.00055 | CD74;TNFRSF1B |
| GO:0042110 | T cell activation | 0.000778 | CD74;HLA-DPB1;TNFRSF1B |
| GO:0002367 | cytokine production involved in immune response | 0.00081 | CD74;TNFRSF1B |
| GO:0002429 | immune response-activating cell surface receptor signaling pathway | 0.000823 | HLA-DRB5;HLA-DPB1;HLA-DRB1 |
| GO:0008630 | intrinsic apoptotic signaling pathway in response to DNA damage | 0.000842 | CD74;TNFRSF1B |
| GO:0051249 | regulation of lymphocyte activation | 0.000885 | CD74;HLA-DPB1;TNFRSF1B |
| GO:0050671 | positive regulation of lymphocyte proliferation | 0.001311 | CD74;HLA-DPB1 |
| GO:0032946 | positive regulation of mononuclear cell proliferation | 0.001331 | CD74;HLA-DPB1 |
| GO:0002700 | regulation of production of molecular mediator of immune response | 0.001496 | CD74;TNFRSF1B |
| GO:0070665 | positive regulation of leukocyte proliferation | 0.001496 | CD74;HLA-DPB1 |
| GO:0042129 | regulation of T cell proliferation | 0.001879 | HLA-DPB1;TNFRSF1B |
| GO:0050728 | negative regulation of inflammatory response | 0.002201 | TNFRSF1B;ACP5 |
| GO:0042098 | T cell proliferation | 0.002601 | HLA-DPB1;TNFRSF1B |
| GO:0050870 | positive regulation of T cell activation | 0.003125 | CD74;HLA-DPB1 |
| GO:1903039 | positive regulation of leukocyte cell-cell adhesion | 0.003628 | CD74;HLA-DPB1 |
| GO:0045657 | positive regulation of monocyte differentiation | 0.004278 | CD74 |
| GO:0051048 | negative regulation of secretion | 0.004307 | CD74;TNFRSF1B |
| GO:0031348 | negative regulation of defense response | 0.004343 | TNFRSF1B;ACP5 |
| GO:0002604 | regulation of dendritic cell antigen processing and presentation | 0.004705 | CD74 |
| GO:0045060 | negative thymic T cell selection | 0.004705 | CD74 |
| GO:0070278 | extracellular matrix constituent secretion | 0.004705 | TNFRSF1B |
| GO:0022409 | positive regulation of cell-cell adhesion | 0.004928 | CD74;HLA-DPB1 |
| GO:0002468 | dendritic cell antigen processing and presentation | 0.005131 | CD74 |
| GO:0002903 | negative regulation of B cell apoptotic process | 0.005131 | CD74 |
| GO:0031392 | regulation of prostaglandin biosynthetic process | 0.005131 | CD74 |
| GO:0043383 | negative T cell selection | 0.005131 | CD74 |
| GO:0045080 | positive regulation of chemokine biosynthetic process | 0.005131 | CD74 |
| GO:0045416 | positive regulation of interleukin-8 biosynthetic process | 0.005131 | CD74 |
| GO:1902337 | regulation of apoptotic process involved in morphogenesis | 0.005131 | TNFRSF1B |
| GO:2000341 | regulation of chemokine (C-X-C motif) ligand 2 production | 0.005131 | CD74 |
| GO:0045059 | positive thymic T cell selection | 0.005558 | CD74 |
| GO:0010935 | regulation of macrophage cytokine production | 0.005984 | CD74 |
| GO:0072567 | chemokine (C-X-C motif) ligand 2 production | 0.005984 | CD74 |
| GO:1903054 | negative regulation of extracellular matrix organization | 0.005984 | TNFRSF1B |
| GO:1904748 | regulation of apoptotic process involved in development | 0.005984 | TNFRSF1B |
| GO:2001279 | regulation of unsaturated fatty acid biosynthetic process | 0.005984 | CD74 |
| GO:0002440 | production of molecular mediator of immune response | 0.006161 | CD74;TNFRSF1B |
| GO:0097193 | intrinsic apoptotic signaling pathway | 0.006287 | CD74;TNFRSF1B |
| GO:0043518 | negative regulation of DNA damage response, signal transduction by p53 class mediator | 0.006411 | CD74 |
| GO:0045073 | regulation of chemokine biosynthetic process | 0.006411 | CD74 |
| GO:0045410 | positive regulation of interleukin-6 biosynthetic process | 0.006411 | CD74 |
| GO:0051044 | positive regulation of membrane protein ectodomain proteolysis | 0.006411 | TNFRSF1B |
| GO:0002739 | regulation of cytokine secretion involved in immune response | 0.006837 | TNFRSF1B |
| GO:0002830 | positive regulation of type 2 immune response | 0.006837 | CD74 |
| GO:0010934 | macrophage cytokine production | 0.006837 | CD74 |
| GO:0032695 | negative regulation of interleukin-12 production | 0.006837 | ACP5 |
| GO:0042033 | chemokine biosynthetic process | 0.006837 | CD74 |
| GO:0050755 | chemokine metabolic process | 0.006837 | CD74 |
| GO:1902166 | negative regulation of intrinsic apoptotic signaling pathway in response to DNA damage by p53 class mediator | 0.006837 | CD74 |
| GO:1903037 | regulation of leukocyte cell-cell adhesion | 0.006935 | CD74;HLA-DPB1 |
| GO:0031643 | positive regulation of myelination | 0.007263 | TNFRSF1B |
| GO:0002902 | regulation of B cell apoptotic process | 0.007688 | CD74 |
| GO:0150079 | negative regulation of neuroinflammatory response | 0.007688 | TNFRSF1B |
| GO:1902165 | regulation of intrinsic apoptotic signaling pathway in response to DNA damage by p53 class mediator | 0.007688 | CD74 |
| GO:0045414 | regulation of interleukin-8 biosynthetic process | 0.008114 | CD74 |
| GO:0061081 | positive regulation of myeloid leukocyte cytokine production involved in immune response | 0.008114 | CD74 |
| GO:0032496 | response to lipopolysaccharide | 0.008129 | TNFRSF1B;ACP5 |
| GO:0051251 | positive regulation of lymphocyte activation | 0.00832 | CD74;HLA-DPB1 |
| GO:0007159 | leukocyte cell-cell adhesion | 0.008465 | CD74;HLA-DPB1 |
| GO:0002374 | cytokine secretion involved in immune response | 0.008539 | TNFRSF1B |
| GO:0002577 | regulation of antigen processing and presentation | 0.008539 | CD74 |
| GO:0042228 | interleukin-8 biosynthetic process | 0.008539 | CD74 |
| GO:0045019 | negative regulation of nitric oxide biosynthetic process | 0.008539 | ACP5 |
| GO:0045655 | regulation of monocyte differentiation | 0.008539 | CD74 |
| GO:1904406 | negative regulation of nitric oxide metabolic process | 0.008539 | ACP5 |
| GO:0002237 | response to molecule of bacterial origin | 0.008758 | TNFRSF1B;ACP5 |
| GO:0003177 | pulmonary valve development | 0.008965 | TNFRSF1B |
| GO:0045061 | thymic T cell selection | 0.008965 | CD74 |
| GO:0002449 | lymphocyte mediated immunity | 0.009207 | CD74;TNFRSF1B |
| GO:0019883 | antigen processing and presentation of endogenous antigen | 0.00939 | CD74 |
| GO:0032928 | regulation of superoxide anion generation | 0.00939 | ACP5 |
| GO:0045723 | positive regulation of fatty acid biosynthetic process | 0.00939 | CD74 |
| GO:0002460 | adaptive immune response based on somatic recombination of immune receptors built from immunoglobulin superfamily domains | 0.009665 | CD74;TNFRSF1B |
| GO:0048714 | positive regulation of oligodendrocyte differentiation | 0.009815 | TNFRSF1B |
| GO:0051043 | regulation of membrane protein ectodomain proteolysis | 0.009815 | TNFRSF1B |
| GO:1902254 | negative regulation of intrinsic apoptotic signaling pathway by p53 class mediator | 0.009815 | CD74 |
| GO:0032102 | negative regulation of response to external stimulus | 0.009872 | TNFRSF1B;ACP5 |
| GO:0001783 | B cell apoptotic process | 0.01024 | CD74 |
| GO:0090023 | positive regulation of neutrophil chemotaxis | 0.01024 | CD74 |
| GO:0060561 | apoptotic process involved in morphogenesis | 0.010664 | TNFRSF1B |
| GO:0002696 | positive regulation of leukocyte activation | 0.010667 | CD74;HLA-DPB1 |
| GO:0071624 | positive regulation of granulocyte chemotaxis | 0.011089 | CD74 |
| GO:0050867 | positive regulation of cell activation | 0.011434 | CD74;HLA-DPB1 |
| GO:0001516 | prostaglandin biosynthetic process | 0.011937 | CD74 |
| GO:0045408 | regulation of interleukin-6 biosynthetic process | 0.011937 | CD74 |
| GO:0046457 | prostanoid biosynthetic process | 0.011937 | CD74 |
| GO:0022407 | regulation of cell-cell adhesion | 0.01194 | CD74;HLA-DPB1 |
| GO:0045785 | positive regulation of cell adhesion | 0.01194 | CD74;HLA-DPB1 |
| GO:0042226 | interleukin-6 biosynthetic process | 0.012361 | CD74 |
| GO:0070229 | negative regulation of lymphocyte apoptotic process | 0.012361 | CD74 |
| GO:1902624 | positive regulation of neutrophil migration | 0.012361 | CD74 |
| GO:0001782 | B cell homeostasis | 0.012785 | CD74 |
| GO:0002828 | regulation of type 2 immune response | 0.012785 | CD74 |
| GO:0046596 | regulation of viral entry into host cell | 0.012785 | CD74 |
| GO:0061082 | myeloid leukocyte cytokine production | 0.012785 | CD74 |
| GO:0090022 | regulation of neutrophil chemotaxis | 0.012785 | CD74 |
| GO:0002724 | regulation of T cell cytokine production | 0.013209 | TNFRSF1B |
| GO:1902230 | negative regulation of intrinsic apoptotic signaling pathway in response to DNA damage | 0.013209 | CD74 |
| GO:1902253 | regulation of intrinsic apoptotic signaling pathway by p53 class mediator | 0.013209 | CD74 |
| GO:0003176 | aortic valve development | 0.013632 | TNFRSF1B |
| GO:0050779 | RNA destabilization | 0.013632 | TNFRSF1B |
| GO:0051085 | chaperone cofactor-dependent protein refolding | 0.013632 | CD74 |
| GO:0032691 | negative regulation of interleukin-1 beta production | 0.014056 | ACP5 |
| GO:1901797 | negative regulation of signal transduction by p53 class mediator | 0.014056 | CD74 |
| GO:0043368 | positive T cell selection | 0.014479 | CD74 |
| GO:0043516 | regulation of DNA damage response, signal transduction by p53 class mediator | 0.014479 | CD74 |
| GO:0042092 | type 2 immune response | 0.014902 | CD74 |
| GO:0002697 | regulation of immune effector process | 0.015244 | CD74;TNFRSF1B |
| GO:0030224 | monocyte differentiation | 0.015325 | CD74 |
| GO:0042554 | superoxide anion generation | 0.015325 | ACP5 |
| GO:0090322 | regulation of superoxide metabolic process | 0.015325 | ACP5 |
| GO:1902622 | regulation of neutrophil migration | 0.015325 | CD74 |
| GO:1903131 | mononuclear cell differentiation | 0.015325 | CD74 |
| GO:0001819 | positive regulation of cytokine production | 0.015627 | CD74;HLA-DPB1 |
| GO:0010614 | negative regulation of cardiac muscle hypertrophy | 0.015748 | TNFRSF1B |
| GO:0045730 | respiratory burst | 0.015748 | CD52 |
| GO:0045923 | positive regulation of fatty acid metabolic process | 0.015748 | CD74 |
| GO:0051084 | 'de novo' posttranslational protein folding | 0.015748 | CD74 |
| GO:1903427 | negative regulation of reactive oxygen species biosynthetic process | 0.015748 | ACP5 |
| GO:1905314 | semi-lunar valve development | 0.015748 | TNFRSF1B |
| GO:1902742 | apoptotic process involved in development | 0.01617 | TNFRSF1B |
| GO:0014741 | negative regulation of muscle hypertrophy | 0.016593 | TNFRSF1B |
| GO:0032692 | negative regulation of interleukin-1 production | 0.016593 | ACP5 |
| GO:0048713 | regulation of oligodendrocyte differentiation | 0.016593 | TNFRSF1B |
| GO:1902229 | regulation of intrinsic apoptotic signaling pathway in response to DNA damage | 0.016593 | CD74 |
| GO:0050727 | regulation of inflammatory response | 0.016998 | TNFRSF1B;ACP5 |
| GO:0002369 | T cell cytokine production | 0.017015 | TNFRSF1B |
| GO:0006458 | 'de novo' protein folding | 0.017437 | CD74 |
| GO:0030890 | positive regulation of B cell proliferation | 0.017437 | CD74 |
| GO:0150077 | regulation of neuroinflammatory response | 0.017437 | TNFRSF1B |
| GO:1903053 | regulation of extracellular matrix organization | 0.017437 | TNFRSF1B |
| GO:0006509 | membrane protein ectodomain proteolysis | 0.017859 | TNFRSF1B |
| GO:0031641 | regulation of myelination | 0.017859 | TNFRSF1B |
| GO:0002791 | regulation of peptide secretion | 0.018008 | CD74;TNFRSF1B |
| GO:0006692 | prostanoid metabolic process | 0.018281 | CD74 |
| GO:0006693 | prostaglandin metabolic process | 0.018281 | CD74 |
| GO:0045687 | positive regulation of glial cell differentiation | 0.018281 | TNFRSF1B |
