# Supplementary material for: Identification of novel prognosis-related genes in the endometrial cancer immune microenvironment
Source: Aging (Albany NY). 2020 Nov 6;12(21):22152–73. doi: 10.18632/aging.104083 (PMC7695382; doi:10.18632/aging.104083)
Supplement: Supplementary Tables 7 and 8 [file aging-12-104083-s004..pdf]

SUPPLEMENTARY TABLES

Supplementary Table 7. Primer sequences for qRT-PCR.

Name	Sequence
CD74	F: CTCCCAAGCCTGTGAGCAAG<br>R: TGACTCTGGAGCAGGTGCAT
CD52	F: CGCTTCCTCTTCCTCCTACTCACC<br>R: TCCGCTTATGTTGCTGGATGCTG
HLA-DPB1	F: CGGATTTCTACCCAGGCAGCATTC<br>R: TACGGATCAGGTTGGTGGACACG
HLA-DRB1	F: AGCGGCGAGTCCATCCTAAGG<br>R: ACCACTCACAGAACAGACCAGGAG
TNFRSF1B	F: CACGCAGCCAACTCCAGAACC<br>R: AGTCGCCAGTGCTCCCTTCAG
ACP5	F: CTTTGTAGCCGTGGGTGACTGG<br>R: CGAGCGATCTCCTTGGCATTGG
HLA-DRB5	F: CACAGTGGAATGGAGAGCACAGTC<br>R: GAGCAGGCCCAGCACAAAGC
GAPDH	F: GCACCGTCAAGGCTGAGAAC<br>R: TGGTGAAGACGCCAGTGGA

Supplementary Table 8. Primary antibodies used for the detection of protein expression.

Name	Manufacturer	Dilution ratio: Western blotting, Immunohistochemistry
CD74	Affinity Biosciences.OH.USA	1:400
CD52	Affinity Biosciences.OH.USA	1:200
HLA-DPB1	Abcam, Cambridge, UK	1:400
HLA-DRB1	Cell Signaling Technology, Inc., Danvers	1:200
HLA-DRB5	Abcam, Cambridge, UK	1:1000
